# With Our Editors

**Published:** 1907-03-15

**Authors:** 


### WITH OUR EDITORS.

**THE ATTAINMENT OF ASEPSIS IN DENTAL PRACTICE.** In a former issue, while alluding to the need for every effort being made to secure asepsis in all dental operations, we called attention to the fact that many operators, while fancying that they are maintaining such a condition, fail through some omission or other to secure this.

This point is again referred to by W. Cass Grayson, in the course of a paper read by him before the North Midland Branch of the British Dental Association, at Chester.

In the course of this he points out that there are cases in dental practice that demand special precautions and others in which special precautions are advisable; but that it will be just as difficult to persuade dentists to take extraordinary precautions in ordinary cases, as it would be to bring about the universal wearing of antiseptic respirators. "Yet," he adds, "in view of the statement that 'a man who takes a walk from the Mansion House to the Marble Arch probably meets with the germ of every disease known to the Registrar-General,' I should not be surprised to learn that the existence of the immunity largely depends on respirators."

This was alluded to also by G. H. Lloyd, in the course of the subsequent discussion, who spoke of a personal experience where the enivitable white coat was worn, each instrument carefully passed through a methylated flame before being used; while the saliva ejector tube had not received even ordinary attention.

The reader of the paper was careful to impress upon his audience that there is considerable difference between the circumstances and risks of surgical operations, and the surroundings and risks of dental work. In this we quite agree with him, but we are hardly prepared to admit that there are no ordinary dental operations performed to-day that depend for their success on such a complete observance of aseptic precautions as characterizes the work of our modern surgeons. Surely there might be abduced, as controverting such an assertion, the modern treatment of teeth with devitalized pulps; and should it be retorted that many such are successfully saved without the attainment of anything like the ideal state, such an argument can be met with the reply that this statement is also true of surgery.

It, is however, true, as he contends, that there are ordinary dental operations now performed which were too risky before the advent of aseptic surgery. More than this, there are, as he points out, many cases in dental practice where true asepsis is quite impracticable. The following is the typical case as he puts it. Given a patient who is, "mad with toothache," and in which extraction is the only remedy. Supposing we find the whole mouth is in a neglected and filthy condition, "would you," he asks, "advocate spending two or three weeks in removing tartar, treating abscess, and doing anything else that might be necessary, in order to place that mouth in as aseptic a state as possible before you extracted the tooth? Would you do this, and allow the patient to suffer agonies all the time? Would you keep him dosed with opium all the time, or would you at once extract the tooth? I think you would at once extract the tooth, and take the risk, trusting, of course, to whatever subsequent treatment seemed good. I think an immediate extraction would be advised at either a general or a dental hospital, where of all places the value of asepsis is fully recognised. You all know that deaths have occurred as the result of extractions under these conditions, but you know they are so rare that you would not consider it advisable to take the extraordinary—under the circumstances—difficult precautions that would be necessary in order to perform as aseptic an operation as possible."

Mr. Grayson sees no necessity for having our operating rooms in a more aseptic condition than the living rooms of our patients. Such things, therefore, as a tiled or even a parquet flooring, tiled walls and ceiling, washable, unupholstered chairs and seats in general, and a surgically hygienic operating chair in particular, do not enter into his scheme. Nor does he insist on washable operating coats for purposes of asepsis. "If washable coats are necessary, why," he asks, "not washable waistcoats and trousers? Also a complete change of every rag of clothing between patients, as well as a bath and a spray down with an antiseptic." "I do not like to see a dentist with a long beard, but I would not advocate being clean shaven especially," he wittily adds, "when it comes to shaving the head."

Many other things insisted on by the ultra-particular appear to him, and a good many others likewise, somewhat inconsistent. It appears, for instance, to him rather strange to sterilise instruments merely because they have been in the mouth, and then to know that our patients put spoons and forks in their mouths, which have certainly not been sterilised between meals, or always used by the same individual. Even medical men, surgeons and dentists, he points out, take meals at hotels and restaurants with light hearts. Our own cooks stir up our food with the spoon they have just tasted it with, to see if it is all right, and we do not concern ourselves very much about the sanitary state of our cook's mouth. It is an astonishing thing to him that an aseptic crank ever dares to ride in a cab or a railway carriage, not to speak of dining at a restaurant or keeping a cook. "We are told," he adds, "that each patient should be given a clean tumbler, and while admitting that an unclean tumbler is just as disagreeable to a patient as it would be to ourselves, at our own tables or at the table of a hotel or restaurant, I wonder if a tumbler that is merely cleaned in warm water and wiped with a cloth that has been used for other tumblers—which is the usual domestic method—is any better than instruments that have only been cleaned in the same way. If all instruments should be sterilised, why not tumblers? Why sterilise such an instrument as a mouth mirror, for instance, and then give the patient an unsterilised tumbler to put to the lips for the purpose or rinsing out the mouth. I see no advantage in jumping out of the way of a railway train to be crushed to death with a steam-roller."

A possible reply to all this is, of course, that there is no reason why tumblers should not be sterilised by the dentist, as he can easily keep a dozen or so in use, and that we should, as far as possible, be in a position to assert on oath and prove, if need be, should any patient be so unfortunate as to contract an initial sore of the mouth, that the care taken in our office in the way of disinfection and sterilisation is such that the disease must have been contracted elsewhere. This point we shall have occasion to revert to again later. It must be remembered that in all probability the saliva of patients in the so-called secondary stage of specific disease is not free from danger as a source of infection, even when no actual lesion of the mouth is discernable.

Another of the expressions of opinion on the part of the reader of the paper strikes us as rather peculiar. After saying in an early part of this that although the exact or original reasons for many ordinary or every-day precautions are unknown to or not bothered about by those who observe them, they exist and are habitually practised by all refined members of society as part of a code of good manners or ordinary decency of conduct, he subsequently suggests that it is somewhat unreasonable that a dentist should be expected to wash his hands every time he blows his nose. "If we spend half our time," he says, "in washing our hands, many patients will think it goes down in the bill, and if we are careful to explain all about germs they may consider that a visit to the dentist is not only an unpleasant but a very risky thing." For our own part, however, we consider it wise for a dentist to leave the room before using his handkerchief, undoubtedly so if he has anything like a cold and has to do it in earnest. After leaving the room on any pretext he should invariably in our opinion, wash his hands before placing them in the patient's mouth, i. e. in anything like good class practice. With further regard to the need of the dentist sterilising everything that he puts into his patient's mouth, we may well give here a nearly parallel instance to that already suggested. It is the practice, we believe, of all dentists, in anything approaching high class practice, to cover the head-rest of the operating chair with a d'oyley, which is changed for every patient. What, it may be asked, is the good of this since the patient, probably, often travels in a railway carriage, &c., and will very possibly rest his head where others have rested theirs? The reply is the same as that previously suggested, viz., that if the patient, and this is quite a likely event in the case of the young, contract ringworm, &c., it will be quite clear that the dentist's office is the last place that can be looked upon as constituting the source of infection.

We quite recognise, nevertheless, that there is a good deal to be said in favor of the main contention of the author of the paper referred to; this being that: "Too much insistence on elaborate details may bring about a discouragement, that will have its expression in a neglect of the more ordinary precautions, and a refuge in the well-known saying, 'One may as well be hanged for a sheep as a lamb.' *The Dental Surgeon*,—England, Feb. 9, 1907.

**DR. G. V. BLACK BANQUETED.** On the afternoon and evening of January 15th 1907, the St. Louis of Society Dental Science paid its respects to Dr. G. V. Black in a banquet and other exercises. We extract the following from the proceeding as printed in the February, 1907, *Western Dental Journal.*

"OUR HONORED GUEST."

Dr. George A. Bowman of St. Louis spoke to this toast as follows:

"I have accepted this commission (the address of welcome), and I assure you, Dr. Black, it gives the greatest pleasure to bid you welcome to this board and to extend the right hand of fellowship of this society and to express its affection for you as an individual, and the appreciation of its members for what you have done for practical dentistry and dental science throughout the world. We can not honor *you*, rather *you* honor us by consenting to be one with us in this hour and on this happy occasion. Little did I think, when in the dim past, you and I made mud pies together, that you would today occupy the very highest niche in the Temple of Dental Fame, and I still making mud pies. (For then my mud and my pies were as good as yours; but you went to work while I played on.) By this work you unconsciously grew into the giant we feel you are to-day—and behold, this hour. Dr. Black's achievement is a life full of usefulness, a name spoken everywhere with affection, a reputation unsullied and brilliant as a star which will scintillate on forever. Dr. Black—our united sentiment is 'Flowers for the living.' Our hands and our hearts are yours; in testimony of which I give you this bunch of beauties and wish you a long life and a happy one." As Dr. Bowman presented to the guest of honor a beautiful bunch of pink carnations, the applause was most enthusiastic.

Dr. G. V. Black, in replying spoke of "The Profession's Progress." Before entering upon this subject he made a few remarks in response to the many kind things that had been said concerning himself, very modestly disclaiming the distinction accredited him by the preceding speakers. Then he took us into his confidence and told us a little of his boyhood days; how as a boy he spent as much time as he could get in the woods. Sometimes with a rifle over his shoulder, but as often without. How he studied the wild animals, their habits and how they burrowed. The birds, and how they nested and reared their young. That this was his recreation and play. Then he began telling us of the first visit he made to St, Louis after he had begun the practice of dentistry. How he came down the Ohio river and then up the Mississippi river to the city. That the first dental society he ever attended was held not very far from the very spot where the Hotel Jefferson now stands. Told of the strong men he met there. This was before the war and before St. Louis had a railroad. He compared the practice of that day with the present. How each operator must needs invent his own methods, and that in so far as mechanics were concerned, that there had not been as great advance as in the scientific side of the profession. He spoke of the needs of greater scientific research, but said it would always be done by the individual rather than associations or societies. Commended the making of the social feature of society gatherings prominent.

He gave great emphasis to the thought of our obligation to our patients. His talk was a most comprehensive review of the subject for the time at his command.

Rev. W. C. Bitting spoke on "Guardians of the Pearley Gates." He said:

I have been so profoundly impressed by the statements of the honored guest of the evening, that I cannot but yield to my feelings, and call attention to some remarkable statements that he has made. It is seldom that one so eminent in the domain of scientific research exhibits the spirit of ethical and social interpretation, and casts into definite form the highest significance of his life's calling. His words are a beautiful tribute of his own manhood, though not designed to be so. Not the least of his attainments is that which he has tonight shown in the discernment of the high significance of his profession. We shall notice first the moral value of his habits of scientific study. How beautiful was the idyl of his boyhood days when, with keen, sharp eyes he began to see things, and to notice facts about birds, animals and flowers, and then he began to think it the meaning of facts. These are the two things which make science. No man who calls himself scientific dare depart from exactness by the breadth of a hair. Facts which are of no value whatever, until we come to know their scientific meaning. Observation gives us facts as the alphabet of science, and classification takes these letters of the alphabet and builds them into words, while interpretation of generalization builds these words into august sentences. Herein lies the reason for such reverence for one's calling as our honored guest has exhibited all his life, and expresses in his response to our greetings.

This is a great sermon, sorely needed to-day. The men of science should be great preachers of the morality and value of exactness and of its contribution to character. The prudential value of exactness is found in its administration of comfort, and the commercial value of exactness is found in the rewards which come to the expert. The demand for this trait is ubiquitous. It is needed by laborers in every realm especially by those whose work is seamy, by all time-servers, by all who work merely for their wages, without regard to the quality of work; by clerks who are careless of relations to the goods they handle, or to books they keep; by charlatan dentists, quack doctors, shyster lawyers, and by ministers who are more interested in traditions than the truth, and in formularies than in facts. I would gladly welcome our distinguished guest to my own pulpit to talk to my young people, and to business men of the moral value of scientific processes; on the tremendous moral significance of genuine scientific investigation. The whole world should add to a passion for facts, the passion of ordering life according to them. The hunger for reality has come upon us. Science, physiology, literature, art, theology and practical life all feel it.

Did you notice also the statement of our guest that schools and professions exist not for the sake of dollars and cents, but for the sake of the service they may render to humanity? To see one deliberately trample under his feet the crown of gold, which so many are eager to wear, and in its place exalt the badge of service to man-kind, is one of the noblest sights that we shall ever behold. You heard his words about the help of the mother during her maternal service. No one can exaggerate what this means to the home, the state, the development of individual life. You likewise heard his words as to the social ideals that should claim every honorable dentist in the performance of his duties. The ethics of evolution have been thought to be cruel. The "survival of the fittest" has been falsely construed to mean that might should wantonly crush weakness. This law of natural selection applies to the life of the individual organism, and relates to its struggle for food, and so for existence. Hence nature was said to be "red in tooth and claw," and the unethical doctrine that it was right to drive the other man to the death was mistakenly imposed upon evolution as a basis. That is animalism in its worst form, and makes all the processes of civilization a huge and disgusting prize fight.

I think it was John Fiske who first emphasized the truth that the perpetuation of the species had also to be considered. That means that there came a time in the life of a parent when it had to be unselfish in the protection of its offspring. When the human species was reached, the period of its infancy was vastly prolonged, and the time of helplessness was protracted far beyond that of any other of the animal species. This meant that the unselfish life was normal to the highest species, the human. Herein we find a physical basis for altruism. This thought science has inbred into all humanity by virtue of its physical history. Selfishness is abnormal. It defies the very traditions of human life, and assaults the entire history of the race. It is indeed a glorious time during which we have come with men of the first rank in scientific investigation, and in the practical arts of life holding up the vision of service to mankind as the ideal for every worker. We need not be afraid of the commercial results of the unselfish life. Mankind, which says "Seek first the kingdom of God and His righteousness and all those things shall be added to you." What we have heard tonight is only a scientific and professional translation of the highest ethical ideal of the Sermon on the Mount.

And did you notice also the beautiful way in which our honored guest exalted the dignity and glory of his profession? To him it is no drudgery. He finds his recreation and vacation in the loftiest side of his vocation. We all know that what the dentist sees, hears, smells and feels in the pursuit of his profession is not enticing. Beneath the sights and sounds and odors is a great conception, not only of service, but of self culture. Every vocation assumes a most dignified position, from this view point. No man succeeds who does not love his work. *Hands can be hired, but brains and hearts must always be given.* Unless conscience, intelligence and enthusiasm are working through one's digits, the results are barren. This is true ot authors, musicians, artists, mechanics, bookkeepers, ministers as well as dentists. We already have too many brainless, conscienceless machines at work in the world. The bag of gold which lies at the end of the rainbow has been sought in vain by peripatetic processes. The end of the rainbow where the bag of gold is found is our workshops, and lies under the eyes of every man, and at the ends of his fingers. It can never be seen except with a vision of the nobility of one's calling, and through the atmosphere of the glory with which everyone should halo his daily duties. We need today to redeem all our professions, and trades, and business pursuits from the smoky, foggy, cloudy pall of commercialism, and to transfigure everyone of them by the sunlight of high heaven's conception of their dignity and importance. There is no difference in honor between D.D., and D.D.S., except the sibilation. One title is as holy as the other. and both should refer to attainments and motives, as well as substance of work. The day has already dawned when every honorable calling in life is seen to be an opportunity for better service and for self-culture. True men and women will forever turn their eyes away from the mere abundance of things, to genuine quality ot life.

I am glad to be here and listen to these three interpretations of scientific life so beautifully given by the guest of the evening, and far more significant than his splendid professional achievements. Let us reverence this verdict of experience, this deliberate judgment of the expert, this summary of the old man who, like the spies sent into Canaan, gives us tonight the fruit of the promised land, into which those of us who are younger and aspiring should yearn to enter.

